# The pharmacokinetics and pharmacogenomics of voriconazole in pediatric Saudi patients: advancing toward precision medicine

**DOI:** 10.3389/fphar.2026.1959131

**Published:** 2026-10-07

**Authors:** Ahmed Albassam, Abeer Alharbi, Hala Al Anizi, Badr Almuzzaini, Waleed Alhussaini, Alaa Aldahri, Amani Kurdi, Laila Alawdah, Sameh Awwad, Sara Almuhisen, Abdullah Alsultan, Majed Aljeraisy

**Affiliations:** 1 Department of Clinical Pharmacy, College of Pharmacy, Prince Sattam Bin Abdulaziz University, Al-Kharj, Saudi Arabia; 2 Pharmaceutical Sciences Department, College of Pharmacy, King Saud bin Abdulaziz University for Health Sciences (KSAU-HS), Riyadh, Saudi Arabia; 3 King Abdullah International Medical Research Center, Riyadh, Saudi Arabia; 4 The Ministry of National Guard Health Affairs (MNGHA), Riyadh, Saudi Arabia; 5 Pediatric Infectious Diseases and Antimicrobial Stewardship, Pediatric Subspeciality Department, Children’s Specialized Hospital, King Fahad Medical City, Riyadh, Saudi Arabia; 6 Clinical Pharmacy Department, Pharmacy Administration, King Fahad Medical City, Riyadh, Saudi Arabia; 7 Department of Clinical Pharmacy, College of Pharmacy, King Saud University, Riyadh, Saudi Arabia

**Keywords:** CYP2C19 polymorphism, pediatric, pharmacogenomics, pharmacokinetics, therapeutic drug monitoring, voriconazole

## Abstract

**Background:**

Voriconazole is a broad-spectrum triazole antifungal. It is used to prevent and treat invasive fungal infections in immunocompromised pediatric patients. Its clinical use is limited due to its nonlinear pharmacokinetics, high variability, and a narrow therapeutic index. Data on pharmacogenomic effects in pediatric patients are limited, particularly in the Saudi population. This study aimed to evaluate voriconazole pharmacokinetics in pediatric patients and to assess the impact of CYP2C19 genotypes on drug concentrations and clearance.

**Methods:**

A prospective observational study was conducted from October 2022 to October 2024 at two tertiary care centers in Riyadh, Saudi Arabia. Saudi pediatric patients aged between 1 and 14 years receiving voriconazole were enrolled after informed consent from guardians. Trough blood samples were collected on the fifth day of therapy and CYP2C19 genotyping (*2, *3, *17) was performed. The metabolizer status was assigned according to the Clinical Pharmacogenetics Implementation Consortium guidelines.

**Results:**

Forty patients were included. The mean age was 67.7 ± 44.1 months, and the mean weight was 17.8 ± 9.5 kg CYP2C19 phenotypes were distributed as follows: normal metabolizers (65%), rapid metabolizers (17.5%), intermediate metabolizers (10%), and ultrarapid metabolizers (7.5%). No poor metabolizers were observed. The mean voriconazole trough concentration was 2.26 ± 2.68 mg/L and the majority (52.5%) of patients had subtherapeutic levels, 35% were within range, and 12.5% were supratherapeutic. In the multivariable analysis, only weight was significantly associated with clearance (*R* ^2^ = 0.14, P-value = 0.02), whereas genotype showed no significant effect (P-value = 0.48).

**Conclusion:**

Voriconazole trough concentrations showed considerable variability among pediatric patients. Thirty-five percent of patients achieved therapeutic trough concentrations at the first trough assessment and no statistically significant association between CYP2C19 polymorphisms and voriconazole trough concentrations was observed in this cohort. However, the small sample size may have limited the detection of genetic influences. These findings support the conclusion that individualized dosing is needed to optimize therapy.

## 1 Introduction

Voriconazole is a second-generation broad-spectrum triazole antifungal agent. It is widely used for prophylaxis and treatment of invasive fungal infections, particularly in immunocompromised pediatric patients. It exerts its antifungal effects by inhibiting the fungal cytochrome P450-dependent 14α-lanosterol demethylase, a key enzyme in ergosterol biosynthesis. This thereby disrupts fungal cell membrane integrity.

Its pharmacokinetics and pharmacodynamics are complex, exhibiting nonlinear kinetics and significant interindividual variability. Voriconazole also possesses a narrow therapeutic index, thus necessitating therapeutic drug monitoring (TDM) to optimize efficacy and minimize toxicity (Warris et al., 2019). Studies in both adult and pediatric populations have demonstrated improved clinical outcomes with TDM. Trough concentrations within the range of 1–5.5 mg/L are associated with enhanced efficacy and safety, as well as improved clinical outcomes. However, concentrations exceeding 5.5 mg/L have been associated with an increased risk of hepatotoxicity, neurotoxicity, and phototoxicity. This emphasizes the need for individualized dosing strategies (Karthaus et al., 2015).

Pediatric patients exhibit greater variability in voriconazole concentrations compared with adults. This is because of higher hepatic clearance and first-pass metabolism during developmental changes that affect drug pharmacokinetics (Gerin et al., 2011; Tucker et al., 2015). Despite the updated 2012 dosing guidelines, therapeutic drug levels (1–5 mg/L) are often not achieved and a study in a Saudi pediatric ICU found that only 33.8% of patients receiving voriconazole achieved therapeutic levels (1–5 mg/L) (Taher et al., 2024). Several other studies in different countries have reported that 50% of pediatric patients failed to reach the target concentrations (Friberg et al., 2012; Hu et al., 2018; Valle-T-figueras et al., 2021).

Voriconazole concentrations are variable and influenced by many factors, including patient age, concomitant drug-drug interactions, and genetic polymorphisms in the cytochrome P450 2C19 (CYP2C19) enzyme. In particular, voriconazole is primarily metabolized by CYP2C19, yielding an inactive metabolite. Genetic variations in CYP2C19 significantly impact metabolic activity, with the *1/*1 genotype representing normal metabolizers, *2/*2 and *2/*9 genotypes representing poor metabolizers, *1/*2, *2/*17, *1/*9, and *9/*9 genotypes representing intermediate metabolizers, 1/*17 representing rapid metabolizer status and *17/*17 representing ultrarapid metabolizer status. (Sim et al., 2006; Scholz et al., 2009). In Saudi Arabia*,* the CYP2C19*17 allele is present at a prevalence of 26.9%, while the CYP2C19*2 allele frequency has been reported to range from approximately 8.2% to 15% in the Saudi population. (Al-Jenoobi et al., 2013; Saeed and Mayet, 2013). The Clinical Pharmacogenetics Implementation Consortium (CPIC) provides dosing recommendations and advocates for tailored dosing strategies or alternative therapeutic agents that are independent of CYP2C19 metabolism for ultrarapid, rapid, and poor metabolizers (Moriyama et al., 2017). Consequently, the interpretation of CYP2C19 genotype effects on voriconazole metabolism is important for the implementation of personalized dosing regimens, particularly within pediatric populations.

There have been limited studies evaluating the pharmacokinetics and pharmacogenetics of voriconazole among the pediatric population. None of these studies have been from Saudi Arabia. This study thus aimed to investigate the pharmacokinetic profile of voriconazole in pediatric patients in Saudi Arabia. In addition, it aimed to examine the impact of CYP2C19 pharmacogenetic variants on voriconazole concentrations.

## 2 Methods

### 2.1 Study design and setting

A prospective, observational, multicenter study was conducted at King Abdullah Specialized Children’s Hospital (KASCH) and King Fahad Medical City (KFMC) in Riyadh, Saudi Arabia, from October 2022 to October 2024.

This study was approved by the Institutional Review Board (IRB) at both sites, King Abdullah Specialized Children’s Hospital (NRC22R/246/05) and King Fahad Medical City (#23–528). The study was funded by the King Abdullah International Medical Research Center (KAIMRC) and co-funded by King Fahad Medical City.

Pediatric patients aged 1–14 years who received voriconazole, either orally or intravenously, for any indication were enrolled in the study. Informed consent was obtained from their legal guardians. According to the hospital formulary and drug therapy guide, the recommended starting dose was 9 mg/kg every 12 h. Data collection included age, weight, gender, height, dosing information, indication, and laboratory results (aspartate aminotransferase [AST], alanine aminotransferase [ALT], albumin, C-reactive protein [CRP], serum creatinine, total bilirubin, blood urea nitrogen [BUN], and alkaline phosphatase [ALP]). Therapeutic drug monitoring (TDM) was performed to evaluate the proportion of patients who achieved the target voriconazole trough concentrations. The therapeutic range was defined as 1.0–5 mg/L (Li et al., 2024). Patients’ trough concentrations were assessed at steady state on the 5th day of voriconazole initiation.

### 2.2 Sample size calculation

The calculated sample size is 45 patients, determined from the expected allele frequencies of the CYP2C19 alleles in our population, based on a prior study (Al-Jenoobi et al., 2013), and from the expected effect of metabolizer status on voriconazole clearance, based on the study by Karlsson et al., 2009. In the study by Al-Jenoobi et al., 13% of patients were rapid or ultra-rapid metabolizers homozygous for CYP2C19*17 or heterozygous, 0.5% were poor metabolizers, and the remainder were normal metabolizers. Because only 0.5% were poor metabolizers, and for simplicity, the calculation assumed two groups: a) rapid/ultrarapid metabolizers (homozygous for CYP2C19*17 or heterozygous, and b) normal metabolizers. The other assumptions were alpha = 0.05, beta = 0.8, a coefficient of variation in drug clearance of 50%, and that normal metabolizers would have 30% lower clearance than rapid/ultrarapid metabolizers.

### 2.3 Pharmacokinetic sample collection and analytical assay

A 2 mL blood sample was drawn on 5th day of therapy. The sample was sent to the pharmacokinetic lab at King Abdullah International Medical Research Center and then centrifuged at 4,000 rpm for 10 min. The separated plasma was stored at −40 °C until analysis.

Voriconazole was quantified using a sensitive and specific LC-MS/MS method. This method was developed in the KAIMRC laboratory in accordance with FDA bioanalytical guidelines (Alhussaini et al., 2023). Separation was achieved using a UPLC BEH C18 column (1.7 µm, 100 × 2.1 mm) (Waters, Ireland) with gradient elution at a flow rate of 0.30 mL/min.

### 2.4 CYP2C19 genotyping and phenotyping

3 mL of whole blood was collected in an EDTA tube. Genomic DNA was extracted using the Qiagen DNeasy kit (Qiagen, Germany) according to the manufacturer’s protocols. DNA purity and quantity were assessed using the Nanodrop™ spectrophotometer and the broad-range (BR) Qubit 3 fluorometer. Genotyping was performed using TaqMan qPCR with a ready-made genotyping assay, following the manufacturer’s protocols. This was done to identify actionable CYP2C19 SNPs, consistent with the CPIC guidelines (Moriyama et al., 2017). Using fluorescence probe assays from Thermo Fisher Scientific, we investigated CYP2C19*2 (rs4244285), *3 (rs4986893), and *17 (rs12248560). Data were analyzed using TaqMan Genotype software. Minor allele frequencies (MAFs) were characterized in our population and Hardy–Weinberg equilibrium was assessed. Phenotypes were assigned solely based on genotypes, in accordance with the CYP2C19 CPIC guidelines (Moriyama et al., 2017).

### 2.5 Statistical analysis and pharmacokinetic/pharmacogenomic analysis

For baseline demographics, continuous variables were summarized as mean ± standard deviation, and categorical variables were reported as frequencies. This study evaluated two primary outcomes. First, we assessed the proportion of patients with therapeutic, subtherapeutic, or supratherapeutic voriconazole trough concentrations. The therapeutic range was defined as 1–5 mg/L (Gerin et al., 2011; Li et al., 2024). We estimated the mean trough concentration and standard deviation (SD) for each trough group. To determine the impact of genotype and other clinical factors on voriconazole elimination, we estimated each patient’s clearance using a Bayesian approach through the NextDose platform (https://nextdose.otago.ac.nz/). Individual clearance was estimated using the “PK Holford 2016 AVG” model implemented in NextDose, a two-compartment model with linear elimination and first-order absorption that incorporates allometric scaling normalized to fat mass. Patient age, weight, height, sex, dose, route, dosing frequency, and the measured trough concentration were entered into NextDose. The observed concentration was combined with the population-model prior to generate posterior Bayesian estimates of individual pharmacokinetic parameters, including clearance. This approach provides a more robust assessment of individual drug elimination than correlating genotype or clinical variables with trough concentrations alone, as it accounts for differences in dosing and patient characteristics that may confound the interpretation of measured concentrations (Aljutayli et al., 2026). The association between clearance and clinical and genetic variables was analyzed using stepwise multiple linear regression. The variables assessed included genotype, age, weight, CRP, ALT, AST, and albumin, with significance set at 0.05. This is based on prior studies that show that these variables can affect voriconazole clearance (Chen J et al., 2022; Takahashi et al., 2022). All statistical analyses and data visualizations were conducted using R software, and the Hardy–Weinberg equilibrium was assessed using the “genetics” package.

## 3 Results

### 3.1 Patient characteristics

A total of 46 pediatric patients were enrolled in the study. Only 40 met eligibility criteria for the final analysis. Two patients were excluded because of inadequate blood sample quality for CYP2C19 genotyping; three were excluded due to undetectable voriconazole trough concentrations and suspected non-adherence based on clinical pharmacist assessment; and one patient was excluded because he was younger than 1 year of age. The mean age was 67.73 ± 44.14 months, and the mean weight was 17.81 ± 9.51 kg. Of the participants, 23 (57.5%) were male, and 17 (42.5%) were female. The route of administration was oral for 26 patients (65%) and intravenous (IV) for 14 patients (35%).

The average loading dose of voriconazole, administered to 24 patients (60%), was 162.70 ± 79.69 mg. The average maintenance dose across all patients was 151.08 ± 70.18 mg. The mean laboratory values were CRP 68.85 ± 93.17 mg/L, AST 56.19 ± 87.94 U/L, ALT 41.18 ± 70.75 U/L, ALP 244.51 ± 240.60 U/L, albumin 35.15 ± 4.5 g/L, and bilirubin 7.01 ± 4.95 μmol/L (Table 1).

**TABLE 1:** Baseline demographic and clinical characteristics of the study population (N = 40).

Parameters	Value
Age (Years)	5.64 ± 3.67
Weight (kg)	17.81 ± 9.51
Gender	23 males (57.5%) 17 females (42.5%)
Treatment indication Prophylaxis Suspected infection Proven infection	17 patients (42.5%) 3 patients (7.5%) 20 patients (50%)
Loading dose Number Mean (mg)	24 patients 162.70 ± 79.69
Maintenance dose Number Mean (mg)	40 patients 151.08 ± 70.18
Administration route	14 (35%) IV 26 (65%) oral
CRP (mg/L)	68.85 ± 93.17
AST (IU/L)	56.19 ± 87.94
ALT (IU/L)	41.18 ± 70.75
Alkaline phosphatase (U/L)	244.51 ± 240.60
Albumin (g/L)	35.15 ± 4.5
Bilirubin (umol/L)	7.01 ± 4.95
Genotype *2/*17 or *1/*2 intermediate metabolizer *1/*1 normal metabolizer *1/*17 rapid metabolizer *17/*17 ultrarapid metabolizer	N (%) 4 (10%) 26 (65%) 7 (17.5%) 3 (7.5%)

### 3.2 Voriconazole trough levels

After the first trough measurement, the mean voriconazole trough concentration was 2.26 ± 2.68 mg/L. A large proportion of patients (52.5%) had trough levels below the therapeutic range (<1 mg/L), 35% were within the therapeutic range (1–5 mg/L), and 12.5% had concentrations above the therapeutic range (>5 mg/L) (Table 2).

**TABLE 2:** Distribution of voriconazole trough concentrations after the first trough measurement.

Trough number	Mean ± SD (mg/L)	Less than the range (<1 mg/L)	Within the range (1–5 mg/L)	More than range (>5 mg/L)
Trough 1	2.26 ± 2.68	52.5%	35%	12.5%

The patients receiving oral voriconazole (n = 26) had a higher mean trough concentration (2.55 ± 3.07 mg/L) than those receiving intravenous therapy (1.73 ± 1.71 mg/L). However, this difference was not statistically significant (Table 3).

**TABLE 3:** Distribution of voriconazole trough concentrations according to administration routes and CYP2C19 genotype.

​	Category (Administration Route/Genotype)	Mean ± SD (mg/L)	Less than the range (<1 mg/L)	Within the range (1–5 mg/L)	More than the range (>5 mg/L)
Trough	Rout				
	Oral n = 26	2.55 ± 3.07	50%	34.61%	15.39%
	IV n = 14	1.73 ± 1.71	57.14%	35.71%	7.14%
Trough	Genotype				
	Intermediate metabolizer (n = 4)	2.45 ± 1.76	25%	75%	-
	Normal metabolizer (n = 26)	2.29 ± 3.00	57.69%	26.92%	15.38%
	Rapid metabolizer (n = 7)	1.955 ± 1.84	42.85%	57.15%	-
	Ultrarapid metabolizer (n = 3)	2.43 ± 3.48	66.67%	-	33.33%

Subtherapeutic concentrations were observed in 50% of patients in the oral group and 57.14% in the intravenous group. Supratherapeutic levels occurred more frequently among patients receiving oral voriconazole (15.39%) than among those receiving intravenous voriconazole (7.14%).

### 3.3 CYP2C19 polymorphisms and voriconazole pharmacokinetics

Most of the patients (65%, 26/40) were normal metabolizers (*1/*1). This was followed by rapid metabolizers (*1/*17) at 17.5% (7/40), intermediate metabolizers (*2/*17 or *1/*2) at 10% (4/40), and ultrarapid metabolizers (*17/*17) at 7.5% (3/40).

The mean voriconazole trough concentration was 2.26 ± 2.68 mg/L. However, it varied by metabolizer phenotype. Normal metabolizers had a mean trough concentration of 2.29 ± 3.00 mg/L; rapid metabolizers, 1.95 ± 1.84 mg/L; and ultrarapid metabolizers, 2.43 ± 3.48 mg/L. Intermediate metabolizers had a mean trough concentration of 2.45 ± 1.76 mg/L (Table 3).

The distribution of voriconazole trough concentrations across the therapeutic range varies by metabolizer phenotype. Among normal metabolizers, 57.70% of patients had subtherapeutic concentrations, 26.92% were within the therapeutic range and 15.40% had supratherapeutic concentrations. Among rapid metabolizers, 42.85% of patients had subtherapeutic concentrations, 57.15% were within the therapeutic range and no patient had supratherapeutic concentrations. Ultrarapid metabolizers showed the greatest variability, with 66.66% of patients having subtherapeutic concentrations, 33.33% having supratherapeutic concentrations and no patients within the therapeutic range. Among intermediate metabolizers, 25% of patients had subtherapeutic concentrations, 75% of patients were within the therapeutic range, and no supratherapeutic concentrations were observed (Table 3). Dose-normalized trough concentrations showed considerable overlap across CYP2C19 phenotypes, with no statistically significant differences detected among the phenotype groups (P-value = 0.80) (Figure 1).

**FIGURE 1:**
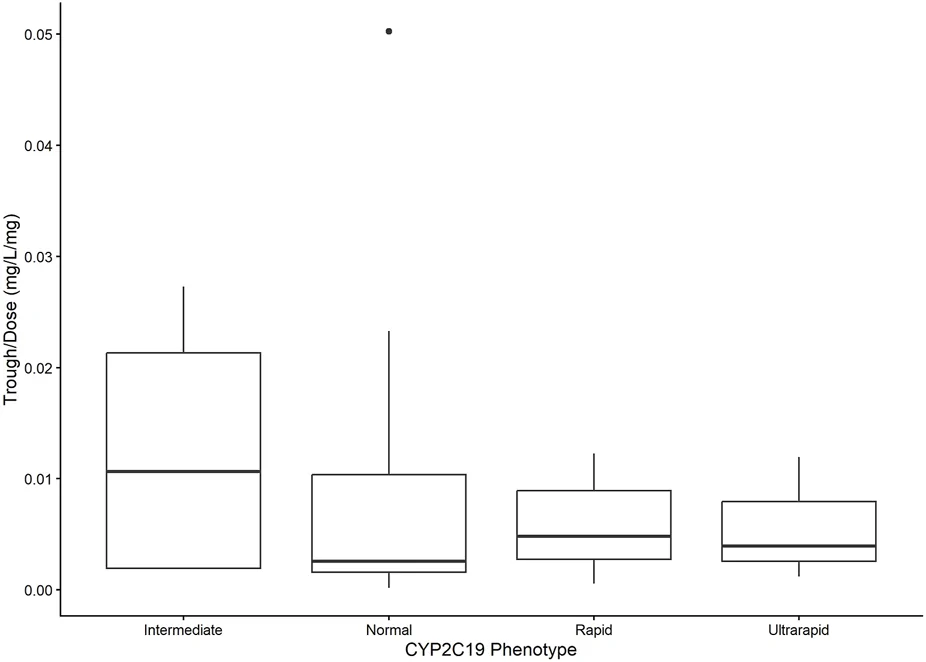
Boxplot of dose-normalized voriconazole trough concentrations stratified by CYP2C19 phenotype.

To evaluate the association between CYP2C19 phenotype and voriconazole elimination, individual voriconazole clearance values were estimated using a Bayesian approach implemented in NextDose software. Due to the small numbers of rapid (RM, n = 7) and ultrarapid (UM, n = 3) metabolizers, these phenotypes were combined into a single group (RM/UM, n = 10) to improve statistical power. Intermediate metabolizers (IM, n = 4) and normal metabolizers (NM, n = 26) were analyzed separately to preserve the biological distinction between reduced and normal CYP2C19 activity. Among the evaluated covariates (age, body weight, sex, and CYP2C19 phenotype), only body weight was significantly associated with voriconazole clearance (*R* ^2^ = 0.149, P-value = 0.02, Table 4). No statistically significant association between CYP2C19 phenotype and voriconazole clearance was observed in this cohort (P = 0.48; Figure 2). The estimated mean clearance was 4.17 ± 3.67 L/h in intermediate metabolizers, 3.84 ± 2.05 L/h in normal metabolizers, and 3.47 ± 1.54 L/h in rapid/ultrarapid metabolizers (Figure 2).

**TABLE 4:** Linear regression analysis of factors associated with voriconazole clearance.

Covariate	β coefficient (L/h)	95% CI	P value
Age, per month	−0.021	−0.058 to 0.017	0.266
Weight, per kg	0.171	0.011 to 0.330	0.037*
CRP, per mg/L	0.002	−0.009 to 0.012	0.751
ALT, per U/L	0.022	−0.038 to 0.082	0.449
AST, per U/L	−0.015	−0.061 to 0.032	0.524
Albumin, per g/L	0.111	−0.072 to 0.295	0.217
CYP2C19	−0.39	−1.16 to 1.94	0.48

* Statistically significant at *P* < 0.05.

**FIGURE 2:**
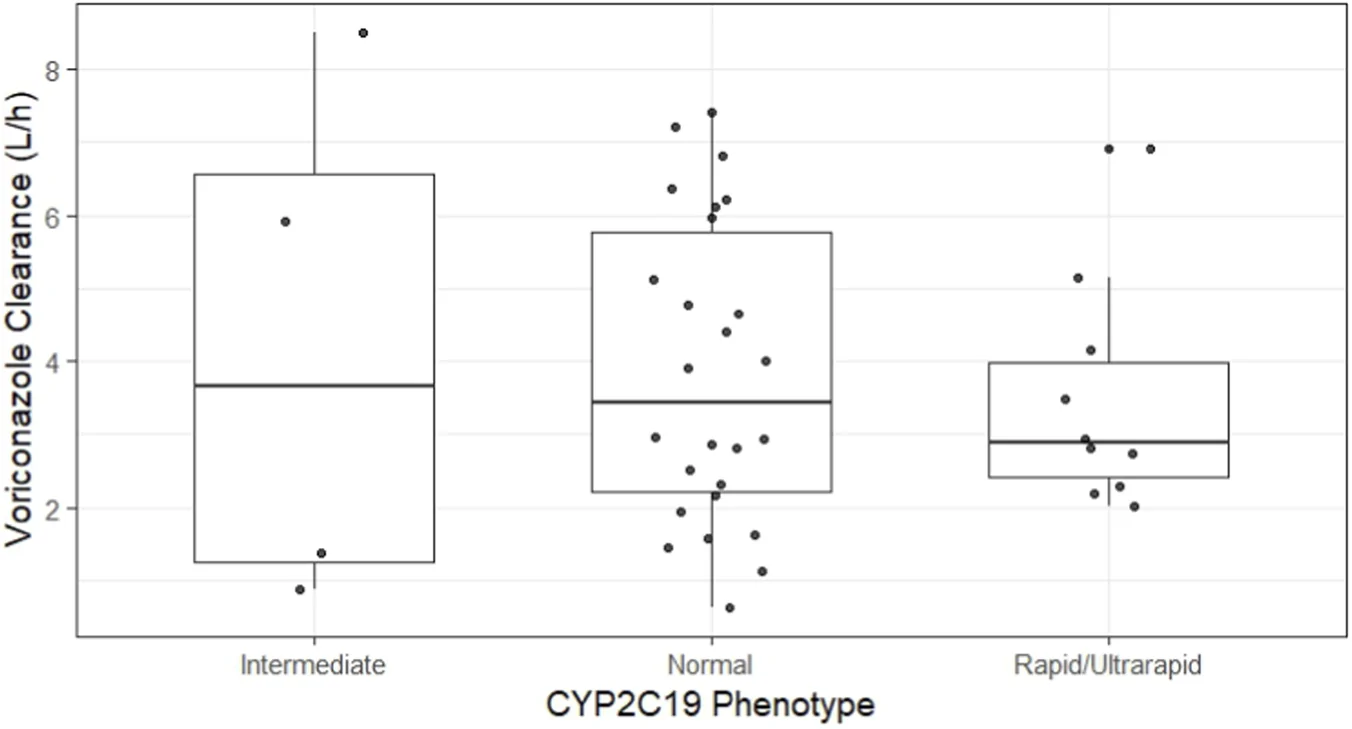
Comparison of voriconazole clearance (L/h) among CYP2C19 phenotypes.

## 4 Discussion

Invasive fungal infections are a major cause of morbidity and mortality among immunocompromised pediatric patients. Voriconazole is effective against fungal infections. However, its pharmacokinetics show substantial interindividual variability (Takahashi et al., 2021; Chen X et al., 2022; Resztak et al., 2024). Data on optimal dosing and the impact of CYP2C19 polymorphisms on voriconazole therapy in pediatric patients remain limited, increasing the risk of treatment failure or toxicity. In our cohort, only 35% of patients achieved trough voriconazole concentrations within the therapeutic range. Conversely, 52.5% were subtherapeutic, and 12.5% were supratherapeutic. No significant significant association between CYP2C19 polymorphisms and voriconazole pharmacokinetics were observed in this cohort.

Voriconazole showed significant inter- and intra-patient variability among pediatric patients. Only 33%–55% of initial trough concentrations fell within the therapeutic range (typically 1–5 or 1–5.5 mg/L) at standard dosing (Boast et al., 2016; Resztak et al., 2021). Similarly, our previous retrospective study of 71 Saudi pediatric patients in the ICU reported that only 33.8% achieved voriconazole trough concentrations within the therapeutic range (1‐5 mg/L). However, 46.5% were subtherapeutic, and 19.7% were supratherapeutic (Taher et al., 2024). Recent studies have shown that a higher percentage of pediatric patients reach target therapeutic concentrations after the initial dose (Lempers et al., 2019; Valle-T-figueras et al., 2021).

The distribution of CYP2C19 allelic variants has been described across different populations. In individuals of European descent, the reported frequencies were *2 (14.6%), *3 (0.02%), and *17 (21.7%) and among East Asian populations, the corresponding frequencies were *2 (28.4%), *3 (5.9%), and *17 (3.8%). In African American populations, the prevalence was *2 (17.5%), *3 (0.09%), and *17 (22.03%) (Ionova et al., 2020). In our cohort, it was CYP2C19 *2 (5%), *3 (0%), and *17 (18.75%) (as illustrated in Supplementary Table S1). These frequencies are similar to previously reported studies of the Saudi population (9.60%, 0.10%, and 25.90%, respectively; see Supplementary Figure S1) (Goljan et al., 2022). These findings highlight interethnic variability in CYP2C19 allele distribution, which will have implications on drug metabolism, therapeutic response, and individualized dosing strategies.

This pharmacogenetic study investigated the effects of actionable CYP2C19 variants (*2, *3, and *17) on voriconazole trough concentrations and clearance. Previous studies demonstrated that voriconazole clearance decreased significantly in CYP2C19 poor metabolizers. This led to higher plasma concentrations compared to normal metabolizers and therefore required lower doses to achieve therapeutic levels (Tian et al., 2021; Hu et al., 2023; Resztak et al., 2024). In our cohort, no CYP2C19 poor metabolizers, defined by biallelic loss-of-function alleles (*2/*2, *2/*3, or *3/*3), were detected. The absence of this phenotype may be explained by the small sample size (N = 40) and the low prevalence of poor metabolizers in the Saudi population, which is reported to be 1.6% (Goljan et al., 2022; Abouelhoda et al., 2023). In our study, no statistically significant differences in voriconazole clearance were observed among normal metabolizers (NM), intermediate metabolizers (IM), and rapid metabolizers/ultra-rapid metabolizers (RM/UM). However, the small size, specifically with IM (n = 4) and RM/UM (n = 10) groups could limited the statistical power to observe modest genotype differences in clearance. Therefore, the lack of a statistically significant association in our cohort should not be interpreted as evidence that CYP2C19 phenotype has no effect on voriconazole clearance. CPIC guidelines recommend an alternative antifungal agent for pediatric patients who are CYP2C19 ultra-rapid or poor metabolizers due to increased risk of under- or overexposure (Moriyama et al., 2017). The absence of poor metabolizers in our study limited our ability to evaluate this genotype-specific recommendation.

The influence of the CYP2C19 genotype on voriconazole clearance may also be attenuated by other genetic or non-genetic factors. These include inflammation, hepatic dysfunction, altered plasma protein binding, variable gastrointestinal absorption, and drug–drug interactions (Li et al., 2024; Coste et al., 2025). These non-genetic factors can contribute to phenoconversion, in which the observed metabolic activity deviates from the genotype-predicted phenotype. This further complicates the assessment of genotype–phenotype correlations. However, this factor was not investigated in our study.

This study has several limitations that should be considered. First, the relatively small sample size may limit the statistical power of the analysis and reduce the generalizability. This could restrict the study’s ability to detect meaningful pharmacogenetic associations with voriconazole pharmacokinetic, specifically among some CYP2C19 phenotype groups. Therefore, the lack of a statistically significant association should not be considered as evidence of no pharmacogenetic effect. However, due to recruitment challenges and limited numbers of patients with certain diseases, small sample sizes are common in pediatric pharmacokinetic studies. Second, the study relied on a single trough concentration measurement per patient, which limited the ability to conduct a full pharmacokinetic analysis. However, this is a common practice in therapeutic drug monitoring and in real-world clinical settings. Finally, as with most observational clinical studies, unmeasured confounders may have altered the results despite efforts to account for the main covariates. Comprehensive data on potentially important factors, including concomitant medications, inflammation, hepatic function, and factors contributing to phenoconversion, were not consistently available and therefore could not be comprehensively evaluated. These confounders, along with underlying disease severity and organ dysfunction, may affect voriconazole metabolism and exposure and pharmacokinetic outcomes.

## 5 Conclusion

This study demonstrates substantial inter-patient variability in voriconazole pharmacokinetics among pediatric patients. Only 35% of patients achieved target therapeutic trough concentrations after the first dose. CYP2C19 polymorphisms are well-known contributors to voriconazole metabolism in adults. However, no statistically significant association between CYP2C19 polymorphisms and voriconazole pharmacokinetics were observed in this cohort. The limited sample size, specifically within the individual phenotype group, may have reduced our ability to detect precise genotype-specific effects. This suggests that the true effect of genetic influence cannot be excluded. As pediatric pharmacotherapy moves toward precision medicine, more studies with larger and more diverse cohorts will be needed to clarify the pharmacogenomic contributions. Meantime, individualized dosing guided by therapeutic drug monitoring and careful clinical judgment remains essential to safe and effective antifungal treatment for children.

## Data Availability

The original contributions presented in the study are included in the article/Supplementary Material, further inquiries can be directed to the corresponding author.
