# Supplementary material for: The pharmacokinetics and pharmacogenomics of voriconazole in pediatric Saudi patients: advancing toward precision medicine
Source: Front Pharmacol. 2026 Oct 7;17:1959131. doi: 10.3389/fphar.2026.1959131 (PMC13683064; doi:10.3389/fphar.2026.1959131)
Supplement: Supplementary file 1 [file Table1.docx]

**Supplementary**

**Supplementary Table 1. Genotype distribution and minor allele frequency (MAF) in pediatric patients (n = 40)**

| SNP (Allele) |  | Genotype | Count (n = 40) | Frequency (%) | MAF | HWE  p-value |
| --- | --- | --- | --- | --- | --- | --- |
| *rs4244285 (*2)* |  | G/G (wild type) | 36 | 90.00% | 0.05 | 0.7392 |
|  |  | G/A (heterozygous) | 4 | 10.00% |  |  |
|  |  | A/A (Homozygous) | 0 | 0.00% |  |  |
| *rs4986893 (*3)* |  | G/G (wild type) | 40 | 100.00% | 0 | 1 |
|  |  | G/A (heterozygous) | 0 | 0.00% |  |  |
|  |  | A/A (homozygous) | 0 | 0.00% |  |  |
| *rs12248560 (*17)* |  | C/C (wild type) | 28 | 70.00% | 0.1875 | 0.0981 |
|  |  | C/T (heterozygous) | 9 | 22.50% |  |  |
|  |  | T/T (homozygous) | 3 | 7.50% |  |  |

**Supplementary Figure 1. Comparative analysis of *CYP2C19* functional variant frequencies in our cohort versus previously reported Saudi population data**
